# *Rhipicephalus* (*Boophilus*) *microplus* aquaporin as an effective vaccine antigen to protect against cattle tick infestations

**DOI:** 10.1186/s13071-014-0475-9

**Published:** 2014-10-12

**Authors:** Felix D Guerrero, Renato Andreotti, Kylie G Bendele, Rodrigo C Cunha, Robert J Miller, Kathleen Yeater, Adalberto A Pérez de León

**Affiliations:** USDA-ARS Knipling Bushland US Livestock Insect Research Laboratory, 2700 Fredericksburg Rd., Kerrville, TX 78028 USA; EMBRAPA Beef Cattle, Avenida Radio Maia, 830-Vila Popular, Caixa postal n. 154, CEP79106-550, Campo Grande, MS Brazil; USDA-ARS Cattle Fever Tick Research Laboratory, 22675 North Moorefield Rd., Edinburg, TX 78541 USA; USDA-ARS Southern Plains Area Office, 1001 Holleman Drive East, College Station, TX 77845 USA

**Keywords:** Cattle tick, Recombinant protein, Vaccine antigen, Aquaporin

## Abstract

**Background:**

Vaccination as a control method against the cattle tick, *Rhipicephalus* (*Boophilus*) *microplus* has been practiced since the introduction of two products in the mid-1990s. There is a need for a vaccine that could provide effective control of *R. microplus* in a more consistent fashion than existing products. During our transcriptome studies of *R. microplus*, several gene coding regions were discovered to encode proteins with significant amino acid similarity to aquaporins.

**Methods:**

A cDNA encoding an aquaporin from the cattle tick, *Rhipicephalus microplus*, was isolated from transcriptomic studies conducted on gut tissues dissected from fully engorged adult female *R. microplus*.

**Results:**

Bioinformatic analysis indicates this aquaporin, designated RmAQP1, shows greatest amino acid similarity to the human aquaporin 7 family. Members of this family of water-conducting channels can also facilitate the transport of glycerol in addition to water. The efficacy of this aquaporin as an antigen against the cattle tick was explored in cattle vaccine trials conducted in Brazil. A cDNA encoding a significant portion of RmAQP1 was expressed as a recombinant protein in *Pichia pastoris*, purified under native conditions using a polyhistidine C-terminus tag and nickel affinity chromatography, emulsified with Montanide adjuvant, and cattle vaccinated intramuscularly. The recombinant protein provided 75% and 68% efficacy in two cattle pen trials conducted in Campo Grande, Brazil on groups of 6 one year old Holstein calves.

**Conclusion:**

The effectiveness of this vaccine in reducing the numbers of adult female ticks shows this aquaporin antigen holds promise as an active ingredient in cattle vaccines targeted against infestations of *R. microplus*.

**Electronic supplementary material:**

The online version of this article (doi:10.1186/s13071-014-0475-9) contains supplementary material, which is available to authorized users.

## Background

The cattle tick, *Rhipicephalus* (*Boophilus*) *microplus*, is an obligate parasitic cattle pest that has established populations throughout the world’s tropical and subtropical regions. *R. microplus* is responsible for significant economic losses to cattle producers because of direct effects through blood loss and damage to hides and indirect effects through diseases it transmits such as babesiosis and anaplasmosis. For example, Grisi et al. [1] estimated Brazil’s annual losses to *R. microplus* parasitism approximate US$3.2 billion. Significant efforts to control this tick are undertaken in most cattle-raising countries and these efforts presently center around the use of acaricides. However, acaricide resistant populations of *R. microplus* have become a major problem in most of the cattle-producing countries of the world and novel cattle tick control technologies are needed to maintain efficiencies in cattle production [2-4].

Vaccination as a tick control method has been practiced since the introduction of two products in the mid-1990s, TickGARD [5] and Gavac^©^ [6], that were developed using the midgut glycoprotein Bm86 as the immunoreactive antigen. TickGARD is no longer commercially available, but Gavac^©^ continues to be used to date, primarily in North and South America. Although the results with Gavac^©^ have been mixed, within integrated tick management systems in some geographic regions, the vaccine has proven to reduce the number of acaricidal applications per year that are required to control *R. microplus* at acceptable levels [7]. As an interesting sidelight to the role of Gavac^©^ in cattle tick control, the product has been shown to provide >99% efficacy against *Rhipicephalus annulatus*, a second cattle tick species which is much less prevalent and invasive than *R. microplus* [8,9]. Nevertheless, the need remains for a vaccine that could provide effective control of *R. microplus* in a more consistent fashion than Gavac^©^. As part of research mining the genome of *R. microplus* for transcripts that would produce effective antigens for a cattle tick vaccine, focused genome [10], transcriptome [11], and proteome [12,13] studies in *R. microplus* have led to identification of genes and gene coding regions that encode proteins with critical functions in the tick [14,15]).

Several of these gene coding regions were discovered to encode proteins with significant amino acid similarity to aquaporins. Aquaporins, originally called water channels, allow the regulation of water transport across the highly hydrophobic lipid bilayer of cell membranes. Members of the aquaporin family have been found in animal taxa from mammals [16] to bacteria [17] and they are very common in certain cell types, with approximately 150,000 protein copies per red blood cell [18]. The structure of the aquaporins is such that two constrictions in the protein act as filters whose selectivity for water, glycerol, urea, and other small molecules is determined by the size and charge of the constriction pore [19]. Because cattle ticks ingest large volumes of blood relative to their body size and weight, they are required to have efficient water transport mechanisms so as to concentrate the blood components for efficient digestion [20]. Thus, the tick aquaporins are critical to tick physiology and appeared a good protein to target as an anti-cattle tick vaccine candidate. The full length transcript for one of the discovered aquaporins, designated RmAQP1, was determined and a fragment of the open reading frame (ORF) was expressed and purified as a recombinant protein in *Pichia pastoris*. This recombinant protein was tested in cattle pen trials for efficacy as a vaccine antigen against *R. microplus*.

## Methods

### Source of tick materials

Ticks that were the source of DNA and RNA for the transcript discovery study were obtained from engorged adult female *R. microplus* of the f20 La Minita strain maintained at The University of Idaho Holm Research Center (Moscow, ID). The La Minita strain was originally collected in 1996 during an outbreak in Starr County, TX and propagated at the USDA-ARS Cattle Fever Tick Research Laboratory in Edinburg, TX. Tissues that were the source of RNA for gene expression study were dissected from 25 1–2 day old adult male and female ticks from the *R. microplus* Deutch strain f41 generation maintained at the Cattle Fever Tick Research Laboratory. The Deutch strain is an organophosphate and pyrethroid susceptible strain originating from an outbreak in 2001 in Webb County, Texas. Tick larvae used in this study to infest cattle for the cattle vaccine trials were obtained from a laboratory colony maintained at EMBRAPA Beef Cattle. The colony was established with *R. microplus* ticks collected from infested cattle in Campo Grande, MS, Brazil. Larvae used for infesting cattle in the vaccine trials were 18 days post-hatch. During the vaccine trials, fully engorged adult female ticks were collected upon host detachment and brought to the laboratory to allow oviposition. Egg masses were incubated in humidity chambers at 28°C and 95% relative humidity to facilitate hatching. Larvae were used for infestation at 18 days after hatching.

### RNA purification, cDNA synthesis & RACE

Total RNA was isolated using the FastPrep-24 Tissue and Cell Homogenizer and Lysing Matrix D (Qbiogene, Irvine, CA, USA) as described in Saldivar et al. [21] from gut tissue dissected from 5 engorged adult female *R. microplus* from the La Minita strain. The total RNA was DNAse treated following manufacturer’s protocol using Turbo DNA-free kit (Ambion, Austin, TX, USA).

One microgram DNase-free total RNA was used to make 5′ and 3′ cDNA using the SMART RACE cDNA Amplification Kit (Clontech Laboratories Inc., Mountain View, CA, USA) and Superscript III Reverse Transcriptase (Life Technologies, Grand Island, NY, USA). Primers (Sigma-Aldrich, The Woodlands, TX, USA) were designed from the available aquaporin-like sequence obtained from GenBank Accession No. CV443183 [11], synthesized, and used to obtain the full length coding region sequence. The target 5′ end was obtained using 20 μL PCR reactions containing 5′ SMART RACE cDNA, Advantage® 2 PCR Enzyme System (Clontech Laboratories Inc.), Universal Primer A and gene specific primer KB-126 (Table 1) according to manufacture’s protocol using the touchdown cycling profile suggested in the SMART RACE protocol booklet. This amplification product was used for a nested PCR using Nested Universal Primer A and gene specific primer KB-126 following manufacturer’s protocol using a cycling profile of thirty cycles including a denaturing step of 94°C for 30 sec and an annealing/extension step of 72°C for 3 min 30 sec. PCR products were analyzed by electrophoresis on 2% SeaKem Gold agarose gels in 1XTBE running buffer (Lonza Rockland, Inc., Rockland, ME, USA) and post-stained using GelStar® Nucleic Acid Gel Stain (Lonza Rockland, Inc.). The detected 965 bp amplicon was excised from the agarose gel and DNA extracted and purified using the QIAquick Gel Extraction Kit (Qiagen, Valencia, CA, USA) according to manufacturer’s protocol. The DNA was concentrated using Pellet Paint Co-Precipitant (Novagen/EMD Chemicals Inc., Gibbstown, NJ, USA), polished, ligated and transformed into XL10 Gold Kan Ultracompetent *Escherichia coli* cells using the PCR Script Amp Cloning Kit (Stratagene/Agilent Technologies Inc., Santa Clara, CA, USA). Individual clones were screened via PCR using internal vector primers and clones producing a correct sized product were used for plasmid DNA preparations with the QIAprep Spin Miniprep Kit (Qiagen) according to manufacturer’s instructions. Plasmid DNAs were sequenced on a 3130xl Genetic Analyzer (Applied Biosystems, Foster City, CA, USA). Identities of each nucleotide in *RmAQP1* were verified on both strands to produce a high quality sequence. Sequences were assembled and analyzed using MacVector with Assembler version 10.0.2 (MacVector Inc., Cary, NC, USA). Basic Local Alignment Search Tools (BLAST) programs were run using multiple BLAST programs available at http://blast.ncbi.nlm.nih.gov/Blast.cgi [22,23]. TOPCONS ([24], http://topcons.net/index.php) was used for the prediction of transmembrane helices in proteins. Net Phos 2.0 Server ([25], http://www.cbs.dtu.dk/services/NetPhos) was used for the predictions of serine, threonine, and tyrosine phosphorylation sites.

**Table 1:** **Primers/Probes used for RmAQP1 transcript cloning and real-time PCR**

**Primer ID**	**Sequence**	**Use**
KB-126	5′ GAGCGGGCACATGCAGTTGTAGGC 3′	Reverse for Aquaporin 5′ RACE
KB-156	5′ ACTCAG**GAATTC**ATGAAGATCGAGAACCT 3′	Forward for insertion into pPICZ αA *EcoRI*
KB-157	5′ TCACTG**GCGGCCGC**CGGGCACATGCAGTTGTAGGC 3′	Reverse for insertion into pPICZ αA *NotI*
KB-238	5′ TCGCCAAAGTGCCGCTATAC 3′	Aquaporin RT-PCR Forward
KB-239	5′ CGTCTTTGTAGGTGGCAAACAC 3′	Aquaporin RT-PCR Reverse
KB-240	5′ 6FAM-CGCCGCACCGACGAAGCCAC-TAMRA 3′	Aquaporin TaqMan Probe
KB-263	5′ TAAGGACCTGTACGCCAACAC 3′	Beta-actin RT-PCR Forward
KB-264	5′ CGGTGATTTCCTTCTGCATACG 3′	Beta-actin RT-PCR Reverse
KB-265	5′ 6FAM-TCTCCGGCGGCACCACCATGTACC-TAMRA 3′	Beta-actin TaqMan Probe
AF018656-363 F	5′ CCTGAGAAACGGCTACCACATC 3′	18S rRNA RT-PCR Forward
AF018656-425R	5′ GTGCCGGGAGTGGGTAATT 3′	18S rRNA RT-PCR Reverse
AF018656-387	5′ 6FAM-AGGAAGGCAGCAGGCGCGC-TAMRA 3′	18S rRNA TaqMan Probe

### Real-time PCR gene expression study

Quantitative PCR studies were designed with the MIQE guidelines in mind [26]. Tissue dissections were performed under phosphate-buffered saline pH =7.4. The tissues dissected from the female ticks were the synganglia, salivary glands, ovaries and midgut while tissues dissected from the male ticks were the synganglia, salivary glands, testes, accessory gland and midgut. Dissected tissues were placed in RNALater (Ambion, Austin, TX, USA) according to manufacturer’s protocol. Total RNA was isolated using the ToTALLY RNA Kit (Ambion) and DNase treated using the Turbo-DNA free kit (Ambion) according to manufacturer’s protocol. The RETROscript Kit Reverse Transcription for RT-PCR (Ambion) was used to produce cDNA from each tissue using 0.1 μg of DNase-free total RNA.

TaqMan probes and primers were designed using Beacon Designer 7.0 (PREMIER BioSoft International, Palo Alto CA; Table 1) and synthesized by Sigma-Aldrich Inc. (The Woodlands, TX, USA) for RmAQP1 and the two reference genes used for normalization, *R. microplus* 18S rRNA gene [21] and beta-actin. Optimization PCRs were run on all three genes to determine optimal reaction conditions, PCR efficiencies, and optimal reagent concentrations. Real-time PCR reactions were carried out in clear low profile 96 well plates (BioRad, Hercules, CA, USA) with microseal film B (BioRad) using 25 μL total volume reactions, which included TaqMan Universal Master Mix No AmpErase UNG (Applied Biosystems Inc.), 250 nM TaqMan probe, tissue specific RETROscript cDNA, and 900 nM forward and reverse primers for all three genes. The cycling profile used on the BioRad CFX96 Real-Time System was 95°C for 10 min, and 50 cycles of 95°C for 15 sec, 60°C for 1 min plus plate read. All samples were run in triplicate and both no-template and no-reverse transcriptase controls were utilized to verify DNA-free status of the samples. The fluorescence emission data analysis for the relative standard curve method for quantification was done using baseline subtracted curve fit mode with CFX Manager Software v1.5 (BioRad).

### Cloning into Pichia pastoris

The partial RmAQP1 ORF used for the vaccine study was amplified with the Advantage® 2 PCR Enzyme System (Clontech Laboratories Inc.) using primers KB-156 and KB-157 (Table 1). The 597 bp amplification product was purified and gel extracted as described above. The RmAQP1 DNA was prepared for ligations by restriction enzyme digestion reactions with EcoRI and NotI (Life Technologies) per manufacturer’s protocol.

The EasySelect *Pichia* Expression Vector (Life Technologies), pPICZ αA restriction enzyme-digested with EcoRI and NotI and purified, was ligated onto the RmAQP1 DNA using the TA Cloning Kit (Life Technologies) using the TA Cloning Kit protocol and 137 ng RmAQP1 insert, 50 ng pPICZ αA EcoRI/NotI digested vector, and 1 unit T4 DNA ligase incubated for 17 hr at 4°C. OneShot TOP10 Electrocomp cells (Life Technologies) were transformed with ligation reaction and plated on low salt LB agar (1% tryptone, 0.5% yeast extract, 0.5% sodium chloride, 1.5% agar) with 25 μg/mL Zeocin^TM^ (Life Technologies). Resulting colonies were screened via PCR using vector primers 5′AOX1 and 3′ AOX1 and DNA isolated from positive colonies using the QIAprep Spin Miniprep Kit (Qiagen) according to manufacturer’s instructions. The sequence of both strands of putative positive clone plasmid DNA was verified by DNA sequencing, followed by analysis with MacVector with Assembler version 10.0.2.

According to the EasySelect *Pichia* Expression Kit protocol, a freshly prepared 80 μL aliquot of electrocompetent *P. pastoris* KM71H strain and 5 μg recombinant expression vector DNA linearized with SstI was used for transformations according to the manufacturer’s instructions using the Bio-Rad Gene Pulser and Pulse Controller at pulse settings of 1.5 kV, 200Ω and 25μFD. Transformation mixtures were plated on YPDS (1% yeast extract, 2% peptone, 2% dextrose, 1 M sorbitol, 2% agar) plates containing 100 μg/mL Zeocin^TM^ and incubated at 30°C for four days to allow colonies to develop.

### Analysis of Pichia pastoris transformants

Direct screening of individual *Pichia* KM71H colonies using PCR was done by modifying the direct screening protocol from Linder et al. [27] and the EasySelect *Pichia* Expression Kit manual with 25 μL reactions using the 5′ and 3′ AOX1 vector primers and 0.16 μL of a 1 vol:1 vol mix of AmpliTaq DNA polymerase (5 U/μL stock; Applied Biosystems) and TaqStart antibody (1.1 μg /μl stock; Clontech). Colonies containing the expected 1,192 bp recombinant product were re-screened using a similar approach but substituting RmAQP1-specific primers.

Selected colonies were Mut phenotyped and small-scale expression experiments used to determine the optimal method and conditions for the expression of the recombinant proteins. These protocols are described in the EasySelect *Pichia* Expression Kit manual for 3 mL cultures grown in BMGY (1% yeast extract, 2% peptone, 100 mM potassium phosphate pH =6.0, 1.34% Yeast nitrogen base with ammonium sulfate without amino acids, 4 × 10^−5^% biotin, 1% glycerol) and BMMY media (BMGY but substituting 0.5% methanol for the 1% glycerol). BMMY cultures were replenished to 0.5% final methanol concentration every 24 hr. Samples were collected at various time points and centrifuged to separate the yeast cells from the culture media supernatant.

Supernatants were frozen in liquid nitrogen and stored at −80°C. Intracellular proteins were purified by a protocol similar to that described in the EasySelect *Pichia* Expression Kit manual. Briefly, 100 μl of breaking buffer (50 mM sodium phosphate pH7.4, 1 mM EDTA, 5% glycerol) +1X FOCUS ProteaseArrest (GBioscience, St. Louis, MO) was used per cell pellet from a 1 ml culture sample. An equal volume of 0.5 mm acid-washed glass beads was added and the sample vortexed for 30 sec and set on ice for 30 sec. A total of 8 vortex/ice cycles were used, the sample frozen at −80°C, thawed and 8 more vortex/ice cycles used before a final short centrifugation to clarify the sample. Samples were concentrated in Amicon Ultracel units (Millipore, Billerica, MA) when necessary.

Both the intracellular cell pellets and the secreted supernatant samples were analyzed by denaturing gel electrophoresis under reducing conditions using the NuPAGE® Electrophoresis System and NuPAGE® 4-12% Bis-Tris gels in the XCell *SureLock*^TM^ Mini-Cell with 1X NuPAGE MOPS SDS Running Buffer (Life Technologies) according to manufacturer’s instructions. Proteins were visualized by staining with Coomassie Brilliant Blue R-250 using a modified Fairbank’s method [28]. Recombinant aquaporin was localized in the cell pellet sample with maximal expression seen after 4 days of induction growth in BMMY.

After the optimal clone and growth conditions were determined, a large scale culture of the clone producing the highest amount of recombinant aquaporin protein was grown in 25 mL BMGY media in 500 ml baffled flasks in a shaking incubator at 30°C to an OD_600_ = 2-6. Cells were harvested by centrifugation and resuspended in BMMY to an OD_600_ = 1 and returned to the incubator for 4 days to induce expression. Every 24 hr, methanol was added to a final concentration of 0.5% to maintain induction and cells were harvested 4 days post-induction. Following centrifugation, the cell pellet was frozen at −70°C until protein extraction.

Total yeast intracellular protein was extracted similarly as described above for the small-scale expression cell pellet protocol with the exception of using 50 mL Breaking Buffer with 1X Protease Arrest and 10 cycles of 30 sec vortexing followed by 30 sec on ice. The cell pellet lysates were then frozen at −70°C overnight and thawed followed by 10 vortex/ice cycles. The protein solution was clarified by centrifugation and the resulting solution concentrated using Centricon Plus-70 Centrifugal Filter Devices (Millipore).

### Purification of expressed recombinant protein

Recombinant protein was purified making use of the 6X-Histidine tag supplied by the vector sequence and the ProBond Purification System (Life Technologies) using ProBond^TM^ nickel-chelating resin under native conditions, initially according to manufacturer’s instructions. We wished to preserve the native protein structure, thus we did not use urea, SDS, or heat in the purification steps. However, the purified protein presented solubility problems upon freezing and thawing and we adapted the ProBond purification steps to utilize buffer (50 mM NaH_2_PO_4_ pH = 8.0, 300 mM NaCl, 2 mM β-mercaptoethanol, 0.4% β -D-1-thioglucopyranoside) plus 10 mM imidazole for binding, the same buffer plus 30 mM imidazole for washing, and buffer plus 300 mM imidazole for elution [29]. Eluted protein was concentrated using Amicon Ultra-15 centrifugation units (Millipore) and, following concentration, the solution was made 50% v/v glycerol and stored at −20°C. This protein solution was used to prepare the vaccine. Protein concentration was quantified by the BioRad Protein Assay Kit I with bovine plasma gamma globulin protein standards, and purity of the protein solution verified by gel electrophoresis as described above using the NuPAGE® Electrophoresis System and NuPAGE® 4-12% Bis-Tris gels in the XCell *SureLock*^TM^ Mini-Cell.

Protein identity was verified by mass spectrometry analysis and Western blotting, taking advantage of the c-*myc* and 6X-His tag epitopes on the recombinant protein that are provided by the expression vector sequence. The WesternBreeze Chromogenic Kit and Anti-*myc*-HRP and Anti-His(C-term)-HRP antibodies (Life Technologies) were utilized with standard protocols provided by the supplier. The supplier-provided alkaline phosphatase-conjugated secondary antibody was utilized to enhance sensitivity. The mass spectrometry analysis was done by Protea Bioscience Group (Morgantown, WV). The recombinant protein (in 50% glycerol solution described above) was purified by 1-D acrylamide gel electrophoresis, extracted from the gel matrix, and digested with trypsin. The resulting peptides were analyzed by LC-MS/MS using an ABSciex5500 Series QTRAP for tandem MS data acquisition followed by a search for peptide matches to the expected sequence of purified antigen.

### Pen trial

Controlled pen trials were conducted to evaluate the immunogenic and protective capacity of the aquaporin antigen adjuvated with Montanide ISA 61 VG (Seppic, Paris) into doses of 2 ml containing 100 μg of the recombinant protein and phosphate-buffered saline (PBS). One-year-old Holstein calves were randomly distributed into groups of six animals each. Negative controls were injected with adjuvant prepared with PBS alone. The animals were injected intramuscularly three times with two week intervals between injections. Serum samples were taken from each animal before the first immunization and weekly thereafter. Twenty-one days after the last injection the animals were challenged with 15,000 larvae of the Campo Grande tick strain. These larvae were delivered in three applications of 5,000 larvae each during one week, placed in separate vials onto the back of the animals. As engorged female ticks detached, they were collected once a day, pooled, and weighed. This sampling was initiated upon the first day detachment started and continued until tick detachment ceased, which was 19 days for Trial 1 and 16 days for Trial 2. Twenty females from each day’s collection were pooled and incubated at 29°C and 85% humidity until egg laying was complete. Eggs collected from each pool were weighed and incubated at 29°C and 85% humidity until hatching was completed to determine the hatch percentage for each pool.

### Bovine serum collection and analysis

Bovine blood was sampled weekly and separated serum frozen until analyzed by ELISA. For the ELISA, sera from all animals in each group were pooled according to day of collection. Microtiter plates were coated with antigen (50 μL per well of a 1 μg antigen/ml solution in 20 mM sodium carbonate buffer, pH 9.6) and incubated overnight at 4°C. Blocking with 2% bovine serum albumin in PBS pH 7.4 containing 0.05% Tween 20 was followed by washing five times with PBS pH 7.4. The plates were incubated for 45 min at 37°C with 100 μL per well of immunized bovine serum diluted to 1:100 in PBS. After washing in PBS pH 7.4, 50 μl of rabbit anti-bovine IgG peroxidase conjugate (Sigma, St. Louis, MO, USA) diluted 1:20,000 was added and the plate incubated for 30 min at room temperature. After washing in PBS pH 7.4, 50 μl of 1.0 mM chromogenic substrate o-phenylenediamine was added and the reaction was stopped after 15 min by adding 50 μl of 0.2 M NaOH. A microplate reader was used to assess the results with absorbance set at 490 nm.

### Efficacy assessment and statistics

Reductions associated with immunization relative to the unvaccinated group were determined for numbers of adult female ticks, egg production, and larval hatching. Vaccine efficacy was calculated as 100 × [1 – (NET × EWPF × H)], where NET, EWPF, and H represent the fraction of the relevant tally in the immunized group relative to that in the control group of the total number of adult female ticks, total weight of eggs per female, and % hatch of eggs, respectively.

### Ethical approval

The La Minita ticks used for the transcript discovery study were reared at The University of Idaho Holm Research Center (Moscow, ID, USA) following protocols approved by the University of Idaho Institutional Animal Care and Use Committee (IACUC). The Deutch ticks used for the gene expression studies were reared at the USDA- ARS Cattle Fever Tick Research Laboratory (Edinburg, TX, USA) with protocols approved by that Laboratory’s IACUC. The cattle vaccine studies were conducted at EMBRAPA Beef Cattle (Campo Grande, MS, Brazil) under protocols approved by the EMBRAPA review board.

## Results

### Aquaporin-like sequences from the cattle tick

**Figure 1:**
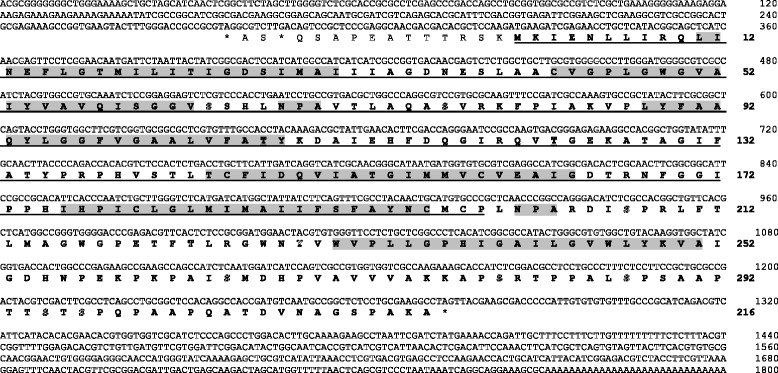
**Nucleotide and translated open reading frame (ORF) sequences for** ***RmAQP1*** **.** Nucleotide sequence numbering starts from the 5′ end of the transcript as determined by RACE. Amino acid numbering begins from the start methionine amino acid. The entire ORF is in bold text, while the portion of the ORF used as the vaccine antigen is underlined. The six predicted transmembrane helices (aa # 11–31, 43–63, 88–108, 144–164, 176–196, and 231–251) and the two NPA motifs (aa # 68–70 and 201–203) are highlighted. The predicted phosphorylation sites at 8 serine (aa # 64, 77, 207, 265, 280, 287, 295, and 297), 3 threonine (aa # 107, 123, and 223), and 1 tyrosine (aa # 229) amino acid residues are indicated in outline font. Multiple in-frame stop codons are present both upstream of the presumptive methionine start and downstream of the stop TAG codon.

Using ESTs from BmiGI Ver 2.0 as a starting point [11], we used 5′ RACE to isolate an 1,800 bp transcript that included the entire ORF to an aquaporin-like protein (Figure 1; GenBank Accession No. KJ626366). As this is the first aquaporin from *R. microplus* to be described, we designated the transcript as *RmAQP1*. The transcript encodes a 216 aa ORF with several stop codons flanking both the N- and C-termini of the presumptive protein, increasing our confidence that we have the authentic ORF. Analysis by TOPCONS (http://topcons.net/index.php) predicted the ORF contains 6 transmembrane helical regions and two NPA motifs, all characteristics of the aquaporin family [30]. The Net Phos 2.0 Server (http://www.cbs.dtu.dk/services/NetPhos) predicted 8 serine, 3 threonine, and 1 tyrosine phosphorylation sites.

**Figure 2:**
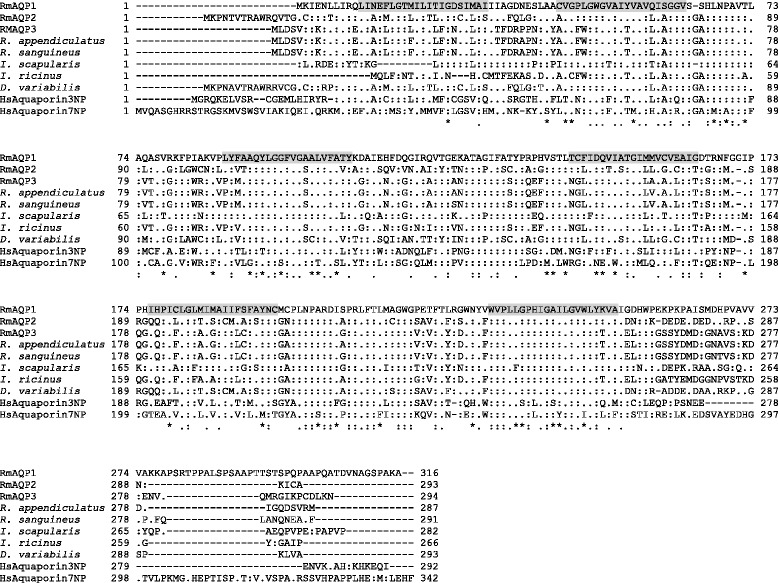
**Amino acid sequence alignments of RmAQP1-3 with putative aquaporins from other tick species.** Alignment was by the ClustalW multiple alignment function of MacVector 12.7.5 using the Gonnet matrix with open gap penalty of 10 and extend gap penalty of 0.05. Determination of amino acid similarity was by chemical properties of amino acid side chains with DE, AGILV, NQ, FWY, RHK, ST, CM, and P comprising the groups considered as conservative substitutions. The accession numbers for the putative tick aquaporins from *R. appendiculatus*, *R. sanguineus*, *I. scapularis*, *I. ricinus*, and *D. variabilis* are CD780384, CAR66115, XP_002399794, CAX48964, and ABI53034, respectively. One member each from the human aquaporin families 3 (NP 004916) and 7 (NP 001161) are also included in the alignment. The RmAQP1 was used as the model for comparing other sequences, with identities indicated by colon (:) and similarities by period (.). In the summary line below the 10 aligned sequences, a colon (:) notes amino acid positions where all 10 sequences contain the identical amino acid, a period (.) indicates all 10 sequences contain identical or similar amino acids, and an asterisk (*) indicates 9 of the 10 aligned sequences have identical or similar amino acids. Gaps inserted to optimize alignments are indicated by a dash (−). The shaded portions of the RmAQP1 sequence indicate the six predicted transmembrane helical regions.

**Figure 3:**
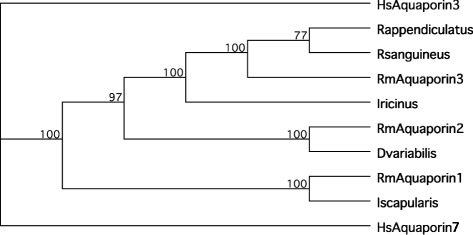
**Phylogenetic tree of putative tick aquaporins.** A phylogenetic tree of the multiple sequence alignment of Figure 2 was determined using the MacVector 12.7.5 package. The analysis was performed using the neighbor joining method using uncorrected distance option and bootstrap mode with 1000 replicates. Tie breaking was set as systematic and gaps distributed proportionally.

We searched other cattle tick transcriptome datasets from ongoing studies in our group and we found 2 other ESTs encoding putative full-length aquaporin-like ORFs, designated RmAQP2 and RmAQP3, and 9 ESTs encoding partial aquaporin-like ORFs (Additional file 1). We produced a ClustalW multiple alignment of RmAQP1-3 with other aquaporin-like ORFs from 5 tick species (Figure 2). The regions that align with the RmAQP1 transmembrane helices 2–6 display more amino acid similarity than other aligned regions. However, the region between predicted transmembrane helices 5 and 6 has a high number of identities in the alignment (Figure 2). In fact, that region between helices 5 and 6 contains 12 of the 40 invariant amino acids that exist over the entire alignment. The phylogenetic tree (Figure 3) that was produced from this alignment showed that RmAQP1 is most similar to the aquaporin from *Ixodes scapularis* (GenBank Accession No. XP_002399794). RmAQP2 was most similar to the aquaporin from *Dermacentor variabilis* (GenBank Accession No. ABI53034), while RmAQP3 was most similar to the aquaporins from *Rhipicephalus appendiculatus* (GenBank Accession No. CD780384) and *Rhipicephalus sanguineus* (GenBank Accession No. CAR66115). In the tissue-specific gene expression study, *RmAQP1* was expressed most highly in the synganglia and lowest in the gut of male ticks, while females expressed *RmAQP1* most highly in the synganglia and lowest in the ovary (Table 2).

**Table 2:** **Gene expression of** ***RmAQP1*** **in various tissues of** ***R. microplus***

**Tissue**	**Relative expression level**	
	**Normalized to 18S**	**Normalized to β-actin**
Adult male ticks		
Synganglia	71	77
Gut	1	1
Salivary gland	4.2	21
Testes	25	14
Accessory gland	12	15
Adult female ticks		
Synganglia	17	9.5
Gut	1	1
Salivary gland	3.0	4.2
Ovary	2.2	0.7

### Production of recombinant aquaporin as vaccine antigen

**Figure 4:**
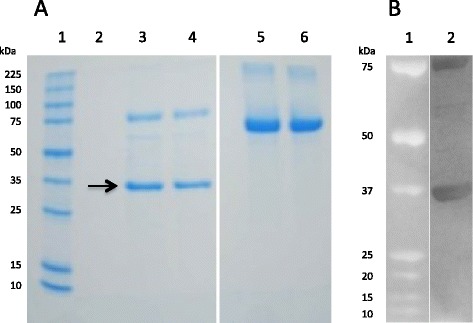
**Gel electrophoresis of recombinant antigen expressed in** ***P. pastoris*** **and analyzed by mass spectrometry and Western blot. A)** Ten μg of purified protein was added to an equal volume of sample buffer (4% sodium dodecyl sulfate, 120 mM Tris pH 6.8, 0.02% bromophenol blue, 5% β-mercaptoethanol), heated for 5 minutes at 90°C, and electrophoresed on a 10% polyacrylamide gel. Following staining with Coomassie G-250 the gel was destained with water for approximately 3 d before the indicated band (arrow) was extracted and analyzed by mass spectrometry. Lane 1: protein molecular weight standards; Lane 2: empty; Lanes 3 and 4: 10 μg of purified vaccine antigen protein with calculated molecular weight of 33.9 kDa (sequence in Additional file 1: Table S1) expressed in *P. pastoris*; Lanes 5 and 6: 10 μg of bovine serum albumin protein standard (MW =66.4 kDa); **B)** Ten μg of purified protein was electrophoresed on a NuPAGE® 4-12% Bis-Tris gel and analyzed by Western blotting using standard protocols provided with the WesternBreeze Chromogenic Kit and Anti-*myc*-HRP antibody (Invitrogen). Lane 1: All Blue Precision Plus Protein Standards (Bio-Rad); Lane 2: Purified recombinant Aquaporin-derived vaccine antigen. The blot image was adjusted through contrast and brightness controls to enable the visualize the minor background products of approximately 60–65 kDa.

During the process of determining the sequence of *RmAQP1*, an opportunity became available for evaluating a vaccine antigen in a controlled cattle pen test. At that time, we had cloned and sequenced only about 600 bp of the transcript, encoding only 91% of what we eventually determined to be the entire ORF (Figure 1). Nevertheless, due to the time-limited nature of the cattle pen test opportunity, we could not complete the transcript cloning within the time constraints and chose to evaluate the existing aquaporin-like antigen as a recombinant protein expressed in *P. pastoris*. The amino acids that comprised the vaccine antigen are shown underlined in Figure 1. The sequence encoding those amino acids was cloned into the *P. pastoris* expression vector pPICZ αA and the resulting recombinant protein purified as described. The full amino acid sequence of the recombinant protein is shown in Additional file 1: Table S1 and has a calculated molecular weight of 33.9 kDa. The first 91 amino acids in the 317 amino acid antigen were from the pPICZ vector, as were the final 27 amino acids. The mass spectrometry analysis verified we had produced the intended protein (Figure 4A). We detected high confidence peptides FSNSTNNGLLFINTTIASIAAK and AQASVRKFPIAK from the vector-provided N-terminus and the RmAQP1-provided ORF, respectively. The Western blot analysis of the purified protein with anti-*myc*-HRP (Figure 4B) and anti-His(C-term)-HRP (data not shown) antibodies confirmed the presence of these moieties on the C-terminus of the antigen.

### Cattle pen tests for aquaporin antigen efficacy against R. microplus

**Figure 5:**
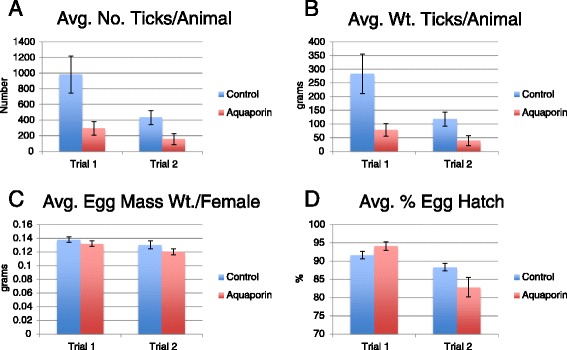
**Tick collection data from cattle stall test vaccine evaluation trials.** For both Trial 1 and Trial 2, means and standard errors are reported for **A)** Average number of ticks collected per animal, **B)** Average weight of ticks collected per animal, **C)** Average egg mass weight per female, and **D)** Average % egg hatch.

**Figure 6:**
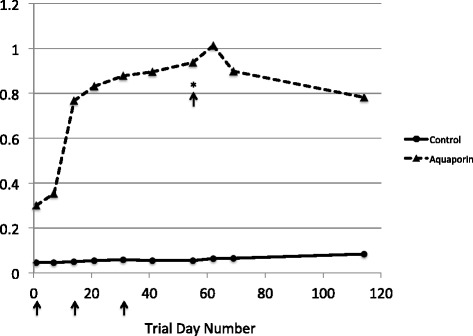
**ELISA results from cattle vaccination trials using recombinant expressed fragment of RmAQP1.** Relative readings from ELISAs are plotted against trial day number for both the cattle group vaccinated with the recombinant expressed fragment of RmAQP1 in PBS plus adjuvant (dashed line and triangles) and the control group vaccinated with PBS plus adjuvant (solid line and circles). Blood was drawn on days 1, 7, 14, 21, 31, 41, 55, 62, 69, and 114 of the test. Three arrows indicate the days of the initial vaccination and the two boosters. The day of larval infestation is noted by an arrow with asterisk.

The recombinant aquaporin-derived antigen was tested with Montanide adjuvant in cattle pen tests. Cattle were vaccinated at the beginning of the test and two and four weeks after the start date. Three weeks after the final immunization, the cattle were challenged with *R. microplus* larvae. The results of the cattle tests are summarized in Figure 5 and detailed in Additional file 2: Table S2, while the efficacy calculation is shown in Table 3. Trial 1 was conducted from September - December 2010 and Trial 2 was conducted from March - July 2011. Bovine blood was sampled weekly from each animal and ELISA results showed that vaccination elicited a specific humoral immune response (Figure 6; Additional file 3: Table S3). The major effect of the aquaporin antigen was on the total tick count resulting from the infestation (Table 3, NET, *P* <0.001). The two trial groups vaccinated with the aquaporin-derived antigen produced 29% of the adult female ticks compared to the control (vaccinated with PBS + adjuvant only). The effects on egg production and egg hatch were minor (*P* >0.05), however the extent of the effect on female tick production resulted in an overall efficacy of 75% and 68% for the two trials (Table 3).

**Table 3:** **Data from cattle stall trials evaluating RmAQP1-derived protein for efficacy as anti-** ***R. microplus*** **vaccine antigen**

**Group**	**Animals**	**Overall tick yield**		**Tests with eggs**			**Hatch**	**NET** ^**a**^	**EWPF** ^**b**^	**H** ^**c**^	**Eff** ^**d**^
	**No.**	**Total no.**	**Total Wt (g)**	**Tick no.**	**Tick Wt (g)**	**Egg Wt (g)**	**%**				
Trial 1											
Control	6	5901	1701.62	334	91.604	46.286	91.4	-	-	-	-
Aquaporin	5	1482	391.625	300	80.865	39.517	93.2	0.25	0.98	1.02	75%
Trial 2											
Control	6	2606	712.16	297	82.69	38.99	88.2	-	-	-	-
Aquaporin	6	956	237.31	236	60.06	28.21	82.9	0.37	0.91	0.94	68%

^a^NET = Reduction in tick numbers = Total number of ticks from the immunized group / Total number of ticks from the control group.

^b^EWPF = Reduction in weight of eggs per female = (Total weight of eggs from the immunized group/ Total number of ticks from immunized group) / (Total weight of eggs from the control group / Total number of ticks from control group).

^c^H = Reduction in hatchability = % hatch from immunized group / % hatch from control group.

^d^Eff = % Overall efficacy compared to control =100 [1-(NET x EWPF x H)].

## Discussion

The efficacy of the aquaporin-derived antigen vaccine against *R. microplus* in our tests was substantial enough to warrant further investigation as a potential control technology against this parasite. Prior to the two pen trials described here, we had conducted a cattle pen trial using a DNA vaccine approach and an expression vector encoding the aquaporin-derived antigen described herein [31]. We obtained approximately 50% efficacy against *R. microplus* (data not shown) while a rBmiTI antigen had approximately 30% efficacy as reported by Andreotti et al. [32]. Additionally, during the vaccination trials reported here, we also had other antigens being evaluated for efficacy against *R. microplus*. For example, in Trial 1 a salivary gland antigen and in Trial 2 a Bm86-Campo Grande antigen was evaluated as a separate group in the pen tests and showed 28% and 49% efficacy, respectively (data not shown). Thus, the aquaporin-derived vaccine was shown to outperform the other vaccines in both our pen trials. The vaccine’s major impact on *R. microplus* was to drastically reduce the yield of adult ticks (Table 3). Effects on average detached female tick weight, average egg mass weight, and hatch were absent or minor (Additional file 2: Table S2).

An aquaporin from *I. ricinus*, IrAQP1 (EMBL Accession Number FN178519), was evaluated for efficacy using *in vivo* feeding assays following dsRNA interference [33]. In contrast to our results, the effects from the IrAQP1 tests were manifested in significant weight reduction in treated ticks, due to reduced blood ingestion. However, reductions in adult tick mortality were not seen. There are a number of reasons that might explain the differences between these aquaporin efficacy tests. There are extensive differences between these two tick species. For example, *I. ricinus* is a three-host tick with an extended life cycle while *R. microplus* parasitizes a single host with a relatively fast life cycle. Also, IrAQP1 and RmAQP1 could be members of different aquaporin families as they have different expression patterns in different tick tissues. *RmAQP1* was expressed most highly in the synganglia of both males and females (Table 2), while *IrAQP1* was not detected in male adult *I. ricinus* or the synganglia of adult female *I. ricinus* [33]. Ball et al. [34] characterized the *RsAQP1* from *R. sanguineus* and found highly similar amino acid sequence and a similar tissue expression pattern as IrAQP1. They described the tick aquaporins as falling into two families based on phylogenetic analysis of the existing aquaporin sequences at the time. Our phylogenetic analysis (Figure 3) maintains the relationships between the aquaporins of *R. appendiculatus*, *R. sanguineus*, *I. ricinus*, and *D. variabilis* noted by Ball et al. [34] with two families of aquaporins noted. However, in our phylogenetic analysis the inclusion of the additional 3 aquaporins from *R. microplus* discovered in our studies and our use of a different *I. scapularis* aquaporin appears to break out an additional aquaporin family that includes *RmAQP1*. This is consistent with our tissue expression results, as *RmAQP1* is the first reported tick aquaporin that has substantial expression in synganglion tissue. We attempted to determine a classification of the *RmAQP1-3* in conjunction with the human aquaporin classifications to perhaps learn more about the aquaporins from *R. microplus*. Using PSI-BLAST with 4 iterations against the NCBI nr protein database entries for *Homo sapiens*, all 3 *R. microplus* aquaporins had highest sequence similarity to several *HsAQP7*-like transcripts, an aquaglyceroporin (data not shown). However, there was also significant similarity to other aquaporin families, including *HsAQP3*, *HsAQP10*, *HsAQP9*, *HsAQP4*. Thus, this approach did not shed much light upon the transport capabilities of the aquaporins of *R. microplus*.

## Conclusion

We have identified 3 aquaporin-like full length ORFs from *R. microplus* transcriptome datasets and a large part of one of those aquaporins, RmAQP1, was discovered to be an efficacious vaccine antigen in Brazilian Holstein calves infested with larvae from the Campo Grande strain of *R. microplus*. Further work is underway to evaluate the general effectiveness of this vaccine in different breeds of cattle and different geographical locations.

## Additional files

Additional file 1: Table S1 Cattle tick aquaporin-like sequences. Additional file 2: Table S2 Vaccine cattle stall test data and efficacy calculations. Additional file 3: Table S3 ELISA data from cattle stall tests of aquaporin anti-cattle tick vaccine.
